# Triglyceride–glucose index and atherogenic index of plasma as predictors of cardiovascular risk in rectal cancer survivors

**DOI:** 10.3389/fcvm.2026.1707899

**Published:** 2026-03-09

**Authors:** Fangmin Shi, Chengnan Liu, Wei Liu, Hao Cai, Qiming Zhao, Chen Zhang, QiYuan Liu, Nan Wu, You Guo

**Affiliations:** 1 Medical Big Data and Bioinformatics Research Centre, First Affiliated Hospital of Gannan Medical University, Ganzhou, China; 2 School of Public Health and Health Management, Gannan Medical University, Ganzhou, China; 3 The First School of Clinical Medicine, Gannan Medical University, Ganzhou, China; 4 Ganzhou Key Laboratory of Medical Big Data, Ganzhou, China

**Keywords:** AIP, cardiovascular disease, plasma proteomics, rectal cancer, TyG

## Abstract

**Background:**

Rectal cancer survivors face elevated cardiovascular disease (CVD) risk, but simple metabolic indices for risk stratification remain underexplored. We investigated associations of triglyceride-glucose index (TyG) and atherogenic index of plasma (AIP) with secondary CVD in rectal cancer patients.

**Methods:**

We analyzed rectal cancer patient data from the First Affiliated Hospital of Gannan Medical University (January 2010 to December 2023) to evaluate the associations between TyG, AIP and CVD. Gene Ontology (GO) and Kyoto Encyclopedia of Genes and Genomes (KEGG) enrichment analyses were performed using UK Biobank proteomics data to identify mediating proteins.

**Results:**

This study included 1,431 cases, among which 1,240 had no CVD and 191 had CVD. Both TyG (OR 1.102, 95% CI: 1.030–1.179, *p* = 0.005) and AIP (OR 1.062, 95% CI: 1.010–1.116, *p* = 0.018) showed independent associations with CVD risk in multivariable models. Age significantly modified these associations (interaction *p* < 0.05), with stronger effects in patients ≥62 years. Combined TyG + AIP modestly improved prediction (IDI: 1.2%, *p* = 0.013) compared to clinical variables alone. Proteomic analysis identified 14 mediating proteins enriched in lipid metabolism, complement/coagulation, and tight junction pathways.

**Conclusions:**

TyG and AIP indices were independently associated with secondary CVD risk in rectal cancer patients, with age-dependent effects. While the predictive improvement was modest, these easily obtainable indices may aid risk stratification when combined with traditional factors. Proteomic findings suggest potential mechanistic pathways warranting further investigation.

## 1 Introduction

Multidisciplinary treatment (MDT) has substantially improved the 5-year survival rate for early-stage rectal cancer to 65%–74% (1–3), but it also increases the risk of cardiovascular disease (CVD) (4, 5). Recent studies indicate that rectal cancer patients face a four-fold elevated risk of developing CVD compared with the general population (6, 7). This elevated risk is largely attributed to oncological treatments, including vascular injury from radiotherapy and metabolic toxicity of chemotherapeutic agents, as well as increased systemic inflammation (8–10). In addition, insulin resistance (IR), a common metabolic disorder, has been shown to promote cancer progression with secondary cardiovascular damage (11, 12).

The triglyceride-glucose index (TyG) and the atherogenic index of plasma (AIP) represent composite indicators reflecting IR and atherogenic dyslipidemia, respectively. Both have demonstrated predictive value for cardiovascular risk in the general population (13, 14). However, their clinical utility in predicting CVD specifically among rectal cancer patients remains unclear, and the mechanisms linking these indices to secondary CVD have not been fully elucidated.

In this study, we investigated the clinical predictive values of TyG and AIP for CVD in rectal cancer patients. We further explored the proteomic mechanisms underlying these associations, aiming to provide novel theoretical insights and practical guidance for clinical risk stratification and personalized management strategies.

## 2 Materials and methods

### 2.1 Study design and study population

We conducted a retrospective cohort study of rectal cancer patients who were initially diagnosed and treated at the First Affiliated Hospital of Gannan Medical University between January 1, 2010, and December 31, 2023. Relevant indicators were measured during the patients' second hospitalization for follow-up examinations (baseline phase), at which point patients with cardiovascular disease had been excluded. Subsequently, patients were followed up for 6 months to 3 years to observe the occurrence of cardiovascular events.

We extracted the following data from the hospital's electronic medical record system, including basic information such as age, sex, and body mass index (BMI), along with blood test indicators including white blood cell count, lymphocytes, neutrophils, eosinophils, basophils, high-density lipoprotein cholesterol (HDL-C), low-density lipoprotein cholesterol (LDL-C), triglycerides (TG), fasting blood glucose (FBG), alanine aminotransferase (ALT), and carcinoembryonic antigen (CEA). We also recorded hyperlipidemia and hyperglycemia status, and calculated TyG and AIP.

**Figure 1:**
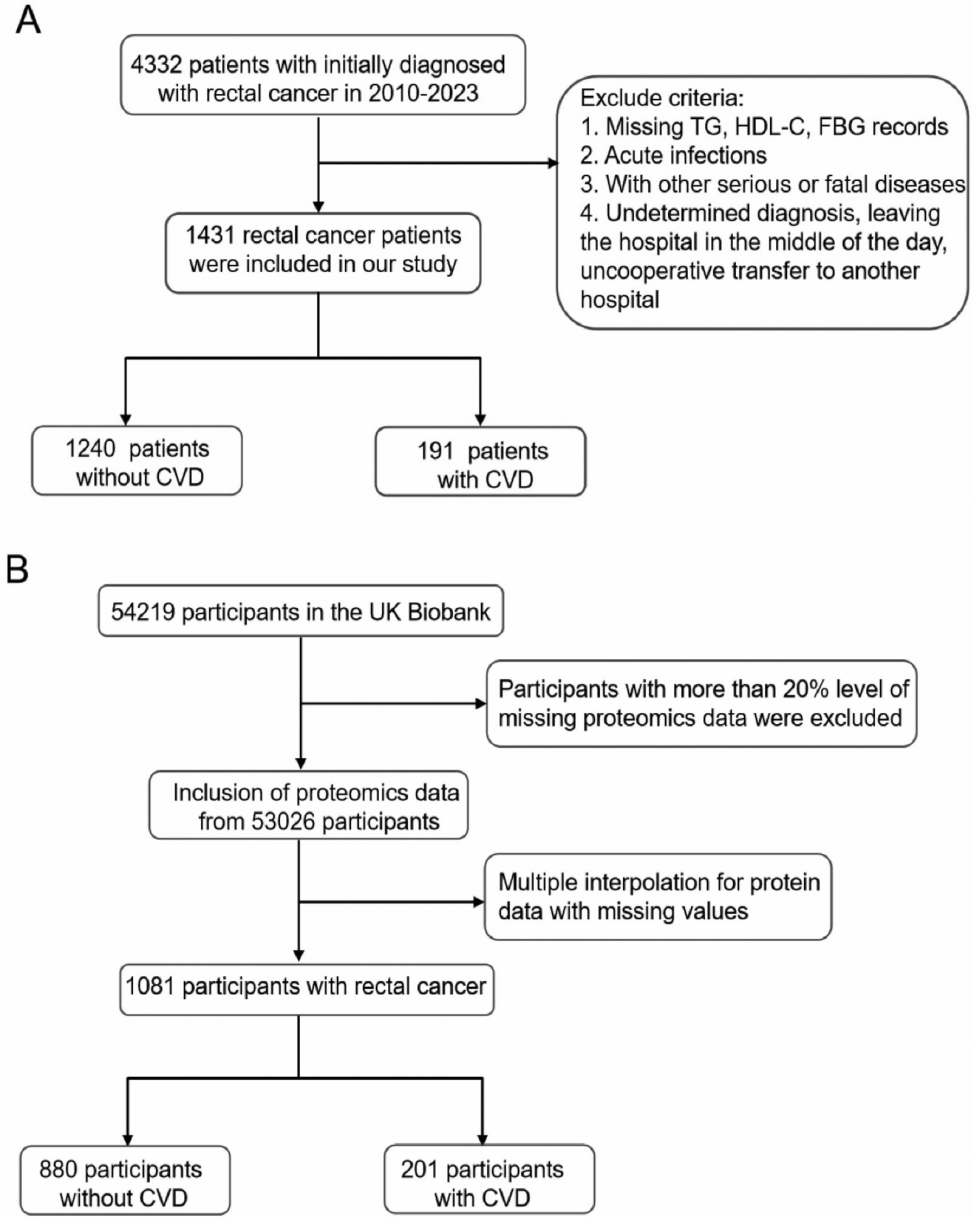
Study population recruitment flowchart. **(A)** Hospital database inclusion and exclusion criteria; **(B)** UKB database inclusion and exclusion criteria.

Figure 1A illustrates our exclusion criteria. For the mediation analysis, we used baseline and proteomics data from the UK Biobank (UKB) as shown in Figure 1B. UKB is a large population-based cohort study in the United Kingdom that recruited approximately 500,000 participants aged 37–73 years between 2006 and 2010 and collected data from questionnaires, physical examinations, and biospecimens (15). From this cohort, blood samples from 54,219 participants underwent high-throughput proteomic profiling, detecting 2,941 protein analytes (2,923 unique proteins). The protocol involved collecting venous blood in EDTA tubes, centrifuging at 2,500 g (4 °C), and storing plasma at −80 °C before transportation to the Olink Analytical Service Center in Sweden. Protein quantification employed the Olink Explore™ Proximity Extension Assay with next-generation sequencing, followed by NPX transformation to normalize data and control for technical variability. After excluding individuals with >20% missing proteomic data, our analysis included 53,026 participants. Within this cohort, we identified 1,081 rectal cancer patients and addressed any remaining missing protein values using mean imputation.

### 2.2 Variable definitions

We enrolled patients diagnosed with rectal cancer according to the International Classification of Diseases, Eleventh Edition (ICD-11) classification, using diagnostic code 2B92. The endpoint was defined as cardiovascular disease complications, which were categorized into nine major types (16): (1) coronary artery disease; (2) heart failure; (3) cardiac arrhythmia; (4) pulmonary arterial hypertension; (5) valvular heart disease; (6) pericardial complications; (7) peripheral vascular disease; (8) stroke; (9) thrombotic disease. According to the Chinese Guidelines for the Prevention and Control of Type 2 Diabetes Mellitus (2020 edition), we defined hyperglycemia as fasting blood glucose ≥7.0 mmol/L, or a history of diabetes mellitus or use of glucose-lowering medication (17). Following the Chinese Guidelines for Lipid Management (2023), we defined hyperlipidemia as meeting any of these criteria: total cholesterol ≥6.2 mmol/L, LDL cholesterol ≥4.1 mmol/L, HDL cholesterol <1.0 mmol/L, or triglycerides ≥2.3 mmol/L (18). We calculated the TyG index using the formula: ln[TG (mg/dL) × FBG (mg/dL)/2] (19), and the AIP index using the formula: log_10_TG (mg/dL)/HDL-C (mg/dL) (20).

### 2.3 Statistical analysis

#### 2.3.1 Descriptive analysis

Continuous variables were expressed as mean ± standard deviation (*x¯* ± *s*). We employed independent samples *t*-tests for normally distributed variables; otherwise, the Mann–Whitney *U* test was used. Categorical variables were expressed as frequencies and percentages [*n*(%)], with chi-square (*χ*^2^) tests for between-group comparisons.

#### 2.3.2 Association analysis of TyG and AIP with cardiovascular disease secondary to rectal cancer

To explore the association between TyG, AIP and CVD secondary to rectal cancer, we first screened relevant variables using univariate logistic regression. Subsequently, we included statistically significant variables from this screening in a multivariate logistic regression model for further analysis. To assess the independent predictive value of TyG index and AIP index, we categorized them into Q1–Q4 groups according to quartiles. We constructed three logistic regression models to further validate their correlation with cardiovascular diseases.

Additionally, to verify whether TyG and AIP indicators differ between colorectal cancer patients with secondary cardiovascular disease and non-cancer patients with primary cardiovascular disease, we conducted a supplementary matched case-control analysis. We collected data on non-cancer patients with primary cardiovascular disease from the same hospital between January 1, 2015, and December 31, 2020. Using the MatchIt package in R software, we performed 1:1 exact matching based on sex, age, and nine categories of cardiovascular disease, successfully matching 175 case-control pairs. This analysis aimed to determine whether rectal cancer patients with secondary cardiovascular disease constitute a cohort with unique metabolic characteristics, thereby providing a basis for developing cancer-specific predictive models.

#### 2.3.3 Dose-response relationship between TyG and AIP and cardiovascular disease secondary to rectal cancer

To further explore the dose-response relationship of TyG index and AIP index with cardiovascular disease secondary to rectal cancer, we performed analyses using a restricted cubic spline model. We selected the number of nodes with the smallest Akaike Information Criterion (AIC) based on our calculations and used age, gender, BMI, hyperglycemia, and hyperlipidemia as covariates.

#### 2.3.4 Subgroup analyses

We used interaction analysis to explore the moderating effects of sex and age on the TyG index and AIP index in relation to the risk of CVD in rectal cancer patients. Since additive interaction analysis is applicable to dichotomous variables (21), we transformed age, TyG index, and AIP index into groups. Using the normal distribution curve intersection method, we determined the optimal thresholds based on the distribution characteristics between patients who did and did not develop CVD. We considered an additive interaction between two factors when the 95% CI for Relative Excess Risk of Interaction (RERI) and Attributable proportion of interaction (AP) did not contain 0 and the 95% CI for Synergy Index (SI) did not contain 1; we identified a multiplicative interaction when the *p* value was less than 0.05. Subsequently, we performed further subgroup analyses on indicators with significant interactions and visualized results using forest plots.

#### 2.3.5 Predictive value of TyG and AIP for cardiovascular disease secondary to rectal cancer

To assess the predictive performance of metabolic indices for secondary CVD risk in rectal cancer patients, we employed a multistage analytical approach. First, we constructed four nested prediction models with increasing complexity: Model 1 (age + CEA), Model 2 (age + CEA + TG), Model 3 (age + CEA + TG + TyG), and Model 4 (age + CEA + TG + TyG + AIP). We evaluated the discriminative ability of each model using receiver operating characteristic (ROC) curves. We further developed a composite index combining TyG and AIP through multivariate logistic regression. This process began with standardizing both indices using z-scores to eliminate scale differences. The weighting coefficients were derived from the absolute values of regression coefficients in the binomial logistic regression model, yielding weights of 65.71% for TyG and 34.28% for AIP. For the predictive performance of composite indices, we compared three metrics: AIC, Bayesian Information Criterion (BIC), and C-statistic.

#### 2.3.6 Mediation analysis

We examined associations of plasma proteins with the TyG and AIP indices in relation to secondary cardiovascular diseases. First, we assessed the relationship between 2923 proteins and each index using multiple linear regression. Next, we performed mediator analysis with Bootstrap validation to determine the mediating effect of significantly associated proteins. Subsequently, we performed Gene Ontology (GO) enrichment analysis using the clusterProfiler package in R, and conducted Kyoto Encyclopedia of Genes and Genomes (KEGG) analysis via the online software KOBAS. Finally, we constructed a protein-protein interaction (PPI) network using the STRING database (https://cn.string-db.org/). We used DrugBank and PharmGKB to analyze mediator proteins for known drug targets.

We conducted all statistical analyses in R 4.2.3 software. Data visualization, including violin and box plots, was created with the ggplot2 package. Statistical significance was defined as a two-sided *p* < 0.05.

## 3 Results

### 3.1 Baseline characteristics

**Figure 2:**
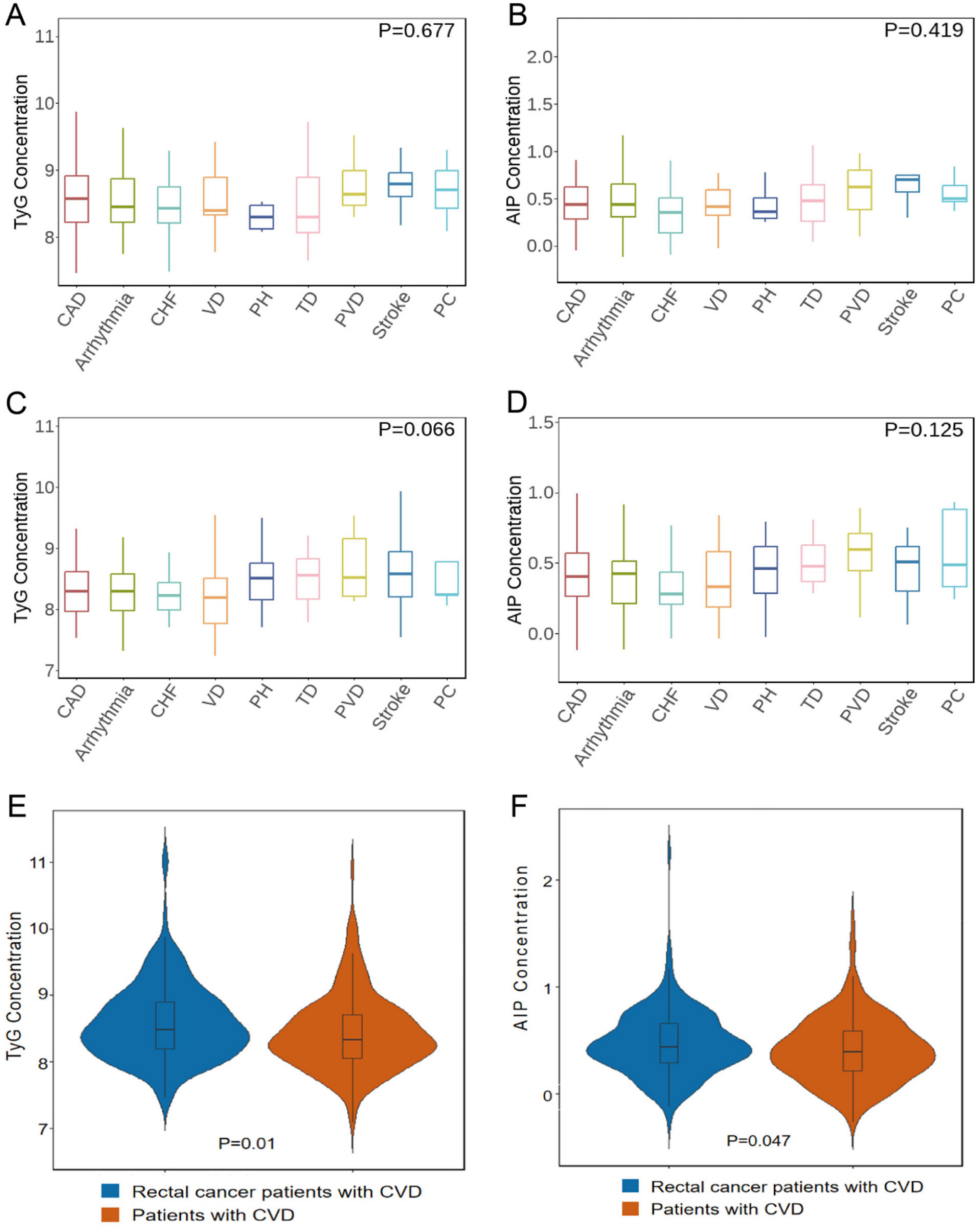
Analysis of intergroup differences in TyG and AIP across cardiovascular disease subtypes. **(A)** TyG expression differences among nine CVD subtypes in rectal cancer patients. **(B)** AIP expression differences among nine CVD subtypes in rectal cancer patients. **(C)** TyG expression differences among nine CVD subtypes in non-cancer CVD patients. **(D)** AIP expression differences among nine CVD subtypes in non-cancer CVD patients. **(E)** TyG comparison between rectal cancer-related CVD and primary CVD. **(F)** AIP comparison between rectal cancer-related CVD and primary CVD.

We included 1,431 rectal cancer patients who fulfilled inclusion criteria (Figure 1). Of these, 1,240 did not develop CVD following treatment and 191 developed secondary CVD. Table 1 summarizes clinical characteristics. Both TyG and AIP differed significantly between groups (TyG: *p* = 0.012; AIP: *p* = 0.022). We also compared TyG and AIP across nine CVD categories in rectal cancer patients with secondary CVD and in patients with primary CVD (Supplementary Table S1). No significant differences in TyG or AIP across the nine categories were observed in either population (Figures 2A–D), supporting the use of a composite CVD endpoint in subsequent analyses.

**Table 1:** Clinical features of rectal cancer patients by CVD presence.

Variable	Without CVD (*n* = 1,240)	With CVD (*n* = 191)	*t/χ*²	*p*-Value
Age (year)	60.9 ± 11.8	65.7 ± 11.1	−5.506	<0.001
Gender			0.001	0.979
Female	469 (37.82)	73 (38.22)		
Male	771 (62.18)	118 (61.78)		
WBC count	6.844 ± 3.574	7.020 ± 3.937	−0.583	0.561
Lymphocyte	1.366 ± 0.569	1.325 ± 0.691	−0.795	0.428
Neutrophil	4.752 ± 3.457	4.956 ± 3.736	−0.710	0.478
Eosinophil	0.168 ± 0.170	0.163 ± 0.142	0.429	0.668
Basophil	0.023 ± 0.018	0.026 ± 0.035	−1.148	0.252
HDL-C	1.063 ± 0.342	1.012 ± 0.312	2.065	0.039
LDL-C	2.621 ± 0.849	2.630 ± 0.997	−0.081	0.935
TG	1.288 ± 0.751	1.426 ± 1.058	−1.736	0.084
FBG	5.398 ± 1.673	5.569 ± 1.664	−1.327	0.186
ALT	20.517 ± 39.754	23.508 ± 49.868	−0.791	0.429
CEA	41.5 ± 199	111 ± 645	−2.522	0.013
BMI	19.929 ± 3.187	19.926 ± 3.299	0.014	0.989
Hyperlipidemia			0.609	0.435
Yes	1,147 (92.5)	173 (90.58)		
No	93 (7.5)	18 (9.42)		
Hyperglycemia			0.001	0.973
Yes	89 (7.18)	13 (6.81)		
No	1,151 (92.82)	178 (93.19)		
TyG	8.468 ± 0.563	8.581 ± 0.575	−2.530	0.012
AIP	0.416 ± 0.291	0.469 ± 0.303	−2.304	0.022

### 3.2 Association of TyG and AIP with CVD among rectal cancer patients

We observed significantly elevated TyG and AIP levels in rectal cancer patients with secondary CVD compared to patients with primary CVD (Figures 2E,F). This difference suggests distinct metabolic profiles between the two groups, underscoring the importance of tailored metabolic risk assessment and clinical staging strategies.

Univariate logistic regression analysis identified several factors associated with secondary CVD to rectal cancer patients, including age, TyG, AIP, TG, and CEA. After adjusting for these potential confounders, multivariate logistic regression analysis confirmed that both TyG (OR = 1.102, 95% CI: 1.03–1.179, *p* = 0.005) and AIP (OR = 1.062, 95% CI: 1.01–1.116, *p* = 0.018) remained significant independent risk factors for secondary CVD in rectal cancer patients. The complete results of both univariate and multivariate logistic regression analyses are presented in Table 2.

**Table 2:** Logistic regression results for TyG and AIP associations with CVD in rectal cancer.

Variable	Univariable		Multivariable	
	OR (95% CI)	*p*-value	OR (95% CI)	*p*-value
Age (year)	1.037 (1.023, 1.052)	<0.001	1.039 (1.024, 1.053)	<0.001
Gender				
Female	1.000			
Male	0.983 (0.719, 1.345)	0.916	—	—
WBC count	1.013 (0.973, 1.054)	0.532	—	—
Lymphocyte	0.883 (0.676, 1.153)	0.359	—	—
Neutrophil	1.057 (0.975, 1.057)	0.453	—	—
Eosinophil	0.834 (0.324, 2.144)	0.706	—	—
Basophil	0.901 (0.802, 1.021)	0.094	—	—
HDL-C	0.633 (0.398, 1.008)	0.054	—	—
LDL-C	1.008 (0.846, 1.201)	0.927	—	—
TG	1.189 (1.014, 1.393)	0.033	1.865 (1.021, 2.917)	0.015
FBG	1.056 (0.973, 1.146)	0.188	—	—
ALT	1.001 (0.998, 1.004)	0.379	—	—
CEA	1.000 (1.000, 1.001)	0.019	1.000 (1.000, 1.001)	0.034
BMI	0.999 (0.953, 1.048)	0.988	—	—
Hyperlipidemia				
Yes	1.000			
No	0.779 (0.459, 1.323)	0.356	—	—
Hyperglycemia				
Yes	1.000			
No	0.945 (0.517, 1.726)	0.853	—	—
TyG	1.419 (1.087, 1.851)	0.010	1.102 (1.030, 1.179)	0.005
AIP	1.860 (1.11, 3.109)	0.018	1.062 (1.010, 1.116)	0.018

Variables marked with “—” were not statistically significant in one-way analysis of variance;1.0 is the reference.

Further stratification by quartiles of TyG and AIP revealed that patients in the highest quartile group exhibited a significantly higher CVD risk compared to those in the lowest quartile group for both indices. These associations remained significant after stepwise adjustment for age, sex, BMI, hyperglycemia, and hyperlipidemia (Supplementary Tables S2, 3). These findings demonstrate a dose-dependent relationship, whereby higher TyG and AIP values are associated with progressively increased CVD risk in rectal cancer patients.

### 3.3 Dose-response relationship of TyG and AIP indices with CVD in rectal cancer patients

We constructed restricted cubic splines models to examine potential nonlinear relationships between metabolic indices and CVD risk. Based on the AIC, we determined that three nodes at the 5th, 50th, and 95th percentiles yielded the optimal model fit. In our models, we adjusted for age, gender, BMI, hyperglycemia, and hyperlipidemia as potential confounders.

**Figure 3:**
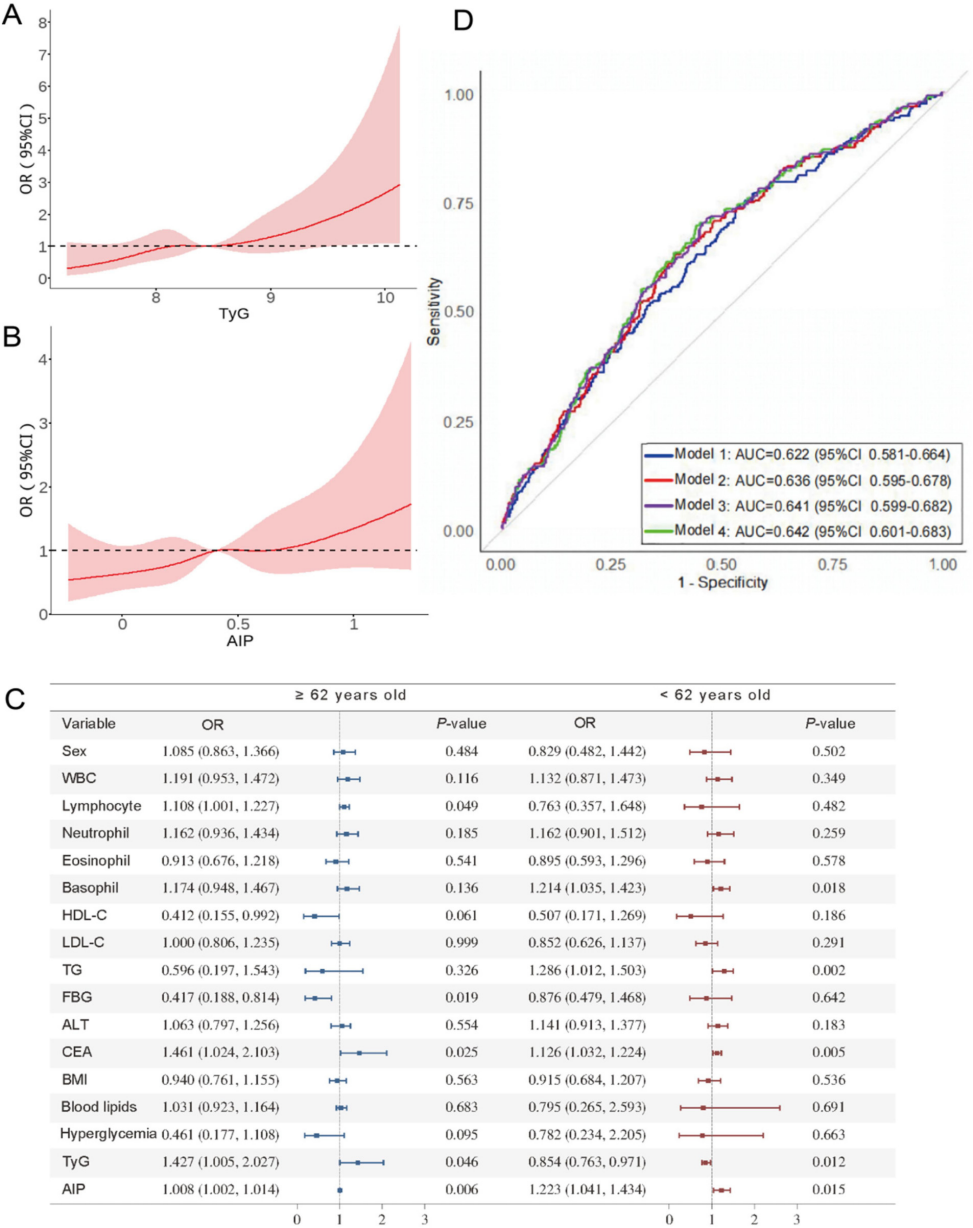
Predictive value of TyG and AIP for CVD in rectal cancer patients. **(A)** Dose-response relationship between TyG and CVD in CRC patients. **(B)** Dose-response plot of AIP vs. CVD in CRC patients. **(C)** Forest plot of subgroup analysis of the association between TyG, AIP and CVD by age. **(D)** Area under the receiver operating characteristic curve (ROC) for prediction models of CVD in rectal cancer patients. Blue line is model 1, including age and carcinoembryonic antigen; red line is model 2, including age, carcinoembryonic antigen, and triglycerides; purple line is model 3, with the addition of TyG to model 2; and green line is model 4, with the addition of AIP to model 3.

Analysis for nonlinear relationships between TyG, AIP, and secondary CVD produced non-significant *p*-values (TyG: *p*-nonlinear = 0.681; AIP: *p-nonlinear* = 0.888), indicating no significant nonlinear associations. As illustrated in Figures 3A,B, we observed a positive linear relationship where the risk of secondary CVD in rectal cancer patients progressively increased with rising TyG and AIP levels. These findings further support the positive relationship between these metabolic indices and cardiovascular risk in the rectal cancer population.

### 3.4 Determination of critical thresholds and interaction effects for CVD risk in rectal cancer patients

We determined the critical threshold values for age, TyG, and AIP by analyzing the intersection points of distribution curves between patients with and without secondary CVD. The following critical thresholds were established: age = 62 years, TyG = 8.58, and AIP = 0.5.

Interaction analyses (Table 3) revealed no significant additive or multiplicative interaction between sex and either TyG or AIP in relation to CVD risk (*p* > 0.05). In contrast, significant additive and multiplicative interactions were observed between age and both metabolic indices (*p* < 0.05). The interaction terms (age & TyG: RERI = 0.973, AP = 0.669; age & AIP: RERI = 0.827, AP = 0.705) indicated that 66.9% and 70.5% of the increased CVD risk in rectal cancer patients could be attributed to the synergistic effect of age with these metabolic indices. In the sensitivity analysis, the results remained consistent using different age cutoffs (Supplementary Tables S4, 5).

**Table 3:** Interaction effects of gender and age on TyG and AIP associations with CVD in rectal cancer patients.

Interaction term	Multiplicative interaction		Additive interaction		
	OR (95% CI)	*p*-value	RERI (95% CI)	AP (95% CI)	SI (95% CI)
Gender&TyG	0.788 (0.417, 1.488)	0.462	−0.292 (-1.366, 0.465)	−0.227 (−1.134, 0.342)	0.494 (−2.135, 3.353)
Gender&AIP	0.672 (0.355, 1.271)	0.221	−0.511 (−1.683, 0.313)	−0.412 (−1.393, 0.223)	0.319 (−1.799, 2.068)
Age&TyG	0.980 (0.963, 0.997)	0.023	0.973 (0.409, 2.317)	0.669 (0.477, 0.861)	2.485 (1.497, 4.125)
Age&AIP	0.677 (0.470, 0.976)	0.037	0.827 (0.791, 0.864)	0.705 (0.454, 0.956)	2.325 (1.321, 4.091)

To further validate the modifying role of age, we performed subgroup analyses stratified by this factor. As presented in Figure 3C, the forest plot demonstrated significant differences in clinical and metabolic parameters associated with secondary cardiovascular disease between participants aged ≥62 years and those <62 years. These findings support the use of age as a reliable stratification criterion for CVD risk assessment in the rectal cancer population.

### 3.5 Predictive value of TyG and AIP for secondary CVD risk in rectal cancer patients

We performed multicollinearity analysis to assess potential multicollinearity among the predictive variables. The variance inflation factors (VIFs) for age, CEA, TG, TyG, and AIP were all below 5. This indicates acceptable levels of independence among these variables.

To evaluate the incremental predictive value of metabolic indices, we developed four nested prediction models: Model 1 (age + CEA), Model 2 (age + CEA + TG), Model 3 (age + CEA + TG + TyG), and Model 4 (age + CEA + TG + TyG + AIP). ROC curve analysis demonstrated that the sequential addition of TyG and AIP to the base model incrementally improved predictive performance for secondary CVD risk in rectal cancer patients (Figure 3D). Specifically, model 4, which combined use of both TyG and AIP, demonstrated superior predictive value compared to models that only incorporated only one or neither of these index. As shown in Table 4, the incorporation of TyG and AIP indices increased the Integrated Discrimination Improvement (IDI) by 1.2% (95% CI 0.3%–2.2%). These findings indicated that TyG and AIP enhanced the diagnostic accuracy for CVD among rectal cancer patients.

**Table 4:** Performance metrics of risk factor models with and without TyG and AIP.

Performance indicators	Risk factor model	Risk factor model + TyG + AIP	*p*-value
IDI	1.000	0.012 (0.003, 0.022)	0.013
AUC	1.000	0.006	0.372

Risk factor model as the base risk factor model included age, carcinoembryonic antigen (CEA) and triglycerides. 1.000 is the reference.

Subsequently, we systematically assessed the goodness of fit for the TyG, AIP, and their composite index model. The resulting standardized composite index was calculated as: 0.6571 × z-TyG + 0.3428 × z-AIP. Where z-TyG and z-AIP represent the standardized values of their respective indices. As shown in Supplementary Table S6, the AIC and BIC analyses revealed that the composite index model (AIC = 1,121.66, BIC = 1,132.19) exhibited a marginal improvement in model fit compared to the standalone TyG model (AIC = 1,122.03, BIC = 1,132.56) and AIP model (AIC = 1,123.01, BIC = 1,133.54). Despite observing this trend, given the modest model improvement (AIC reduction of only 0.37), in actual clinical practice, particularly in resource-constrained medical environments, utilizing the more straightforward single TyG index may be more practical and economical.

### 3.6 Proteomic mediators between metabolic indices and CVD in rectal cancer patients

We analyzed proteomics data from 1,081 rectal cancer patients in the UKB-PPP program to identify potential mediating proteins linking metabolic indices and CVD. Using multiple linear regression, we examined associations between 2,923 plasma proteins and CVD risk in relation to TyG and AIP.

**Figure 4:**
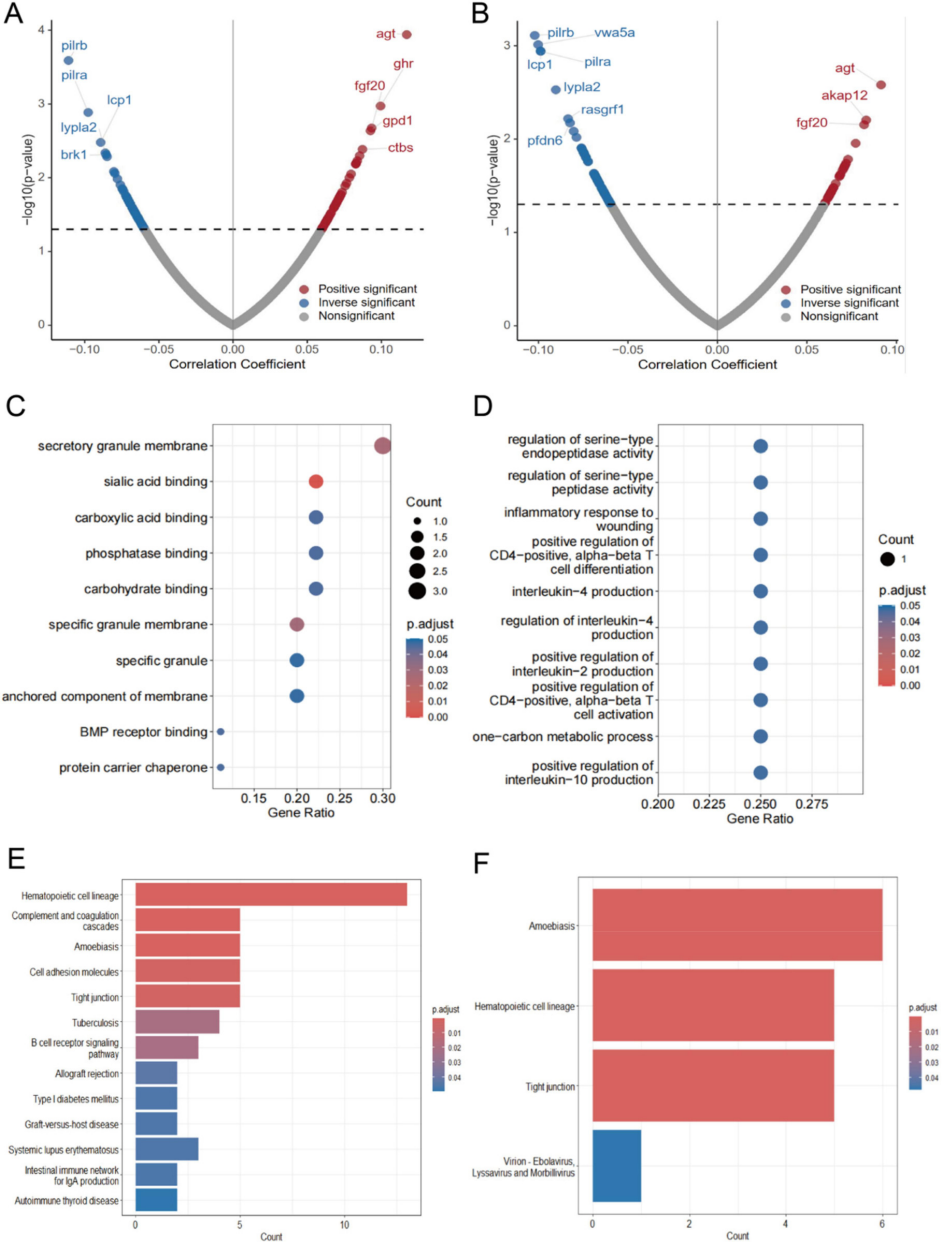
Intermediary protein enrichment analysis of TyG and AIP with CVD. **(A)** Volcano plots of associations between TyG and 2923 proteins in patients with rectal cancer suffering from CVD. **(B)** Volcano plot of association between AIP and 2923 proteins in rectal cancer patients with CVD. **(C)** GO analysis of mediator proteins in TyG-associated proteins. **(D)** GO analysis of mediator proteins in AIP-associated proteins. **(E)** KEGG analysis of TyG intermediary protein PPI networks. **(F)** KEGG analysis of PPI networks of AIP mediator proteins.

For the TyG index (Figure 4A), 109 proteins were identified significantly associated with CVD. Among these, 54 proteins showed positive correlations, with AGT, GHR, and FGF20 being the most significant. While 55 proteins exhibited negative correlations, most notably PILRB, PILRA, and LYPLA2. Mediation analysis revealed 10 proteins that significantly mediated the TyG-CVD relationship (Supplementary Figure S1). Gene Ontology analysis of these 10 mediating proteins showed enrichment in functional categories including sialic acid binding, secretory granule membrane, and specific granule membrane (Figure 4C). KEGG pathway enrichment analysis (Figure 4D) highlighted three major pathways: hematopoietic cell lineage, complement and coagulation cascades, and amoebiasis. We constructed a PPI network of these mediators using the STRING database (Supplementary Figure S3A). Within this mediator group, seven proteins demonstrated significant suppressive effects: BMP6 (−4.0%), CD22 (−4.8%), CD83 (−5.5%), CYTH3 (−6.7%), LAMP3 (−3.6%), PLAUR (−7.7%), and SIGLEC10 (−5.9%).

For the AIP index (Figure 4B), 90 proteins were significantly associated with CVD. Of these, 29 showed positive associations, with AGT, AKAP12, and FGF20 demonstrating the strongest statistical significance. The remaining 61 proteins exhibited negative associations, with PILRB, VWA5A, and LCP1 being the most significant. Mediation analysis identified four proteins that significantly mediated the AIP-CVD relationship (Supplementary Figure S2). Their PPI network is shown in Supplementary Figure S3B. KEGG pathway enrichment analysis (Figure 4F) revealed three main pathways: amoebiasis, hematopoietic cell lineage, and tight junction. Among the four identified mediator proteins, only CA11 demonstrated a substantial masking effect (−4.9%; Supplementary Figure S2).

Finally, using DrugBank and PharmGKB, we evaluated whether the identified mediator proteins were known drug targets (Supplementary Table S7). Our analysis revealed that DCUN1D2, CD177, CYTH3, SPINK6, and CA11 represented unexplored regions in current drug target development, suggesting potentially novel therapeutic avenues for reducing CVD risk in rectal cancer patients.

## 3 Discussion

In This study, we demonstrated for the first time an association between elevated TyG and AIP and secondary CVD development in rectal cancer patients. Furthermore, our proteomic analysis revealed the underlying plasma protein networks connecting these metabolic markers with CVD, thereby elucidating key biological pathways and potential targets for intervention.

Our findings demonstrate a robust association between elevated TyG and AIP indices and incident CVD in rectal cancer patients, consistent with previous studies in the general population (22, 23). The improved predictive capability for secondary CVD achieved by incorporating these indices into risk models further highlights their clinical value. We observed no significant sex-based differences in the relationship between these metabolic indices and CVD risk, consistent with findings by Yang and Lumu et al. (24, 25). Although numerous studies have demonstrated intrinsic sex-based differences in metabolic regulation (26), the complex metabolic syndrome prevalent in cancer patients (27), may attenuate the protective effects of sex hormones, particularly estrogen, on the cardiovascular system. In contrast, our study identified age as a key modifier in the relationship between TyG, AIP and CVD, with significantly enhanced cardiovascular risk in rectal cancer patients older than 62 years. This age-dependent effect likely results from vascular aging and increased endothelial dysfunction due to degradation of elastic fibers and increased collagen deposition in large arteries (28). Additionally, age-related chronic low-grade inflammation plays an important role, not only directly worsening insulin resistance (29) but also accelerating atherosclerosis through sustained activation of pro-inflammatory factors such as IL-6 and TNF-α (30–32).

Concurrently, we discovered that in the population under 62 years old, the TyG index exhibited potential protective effects. This may arise from younger individuals' superior metabolic adaptability and insulin regulatory capacity (33). However, as age progresses, particularly beyond 62 years, the TyG index transforms into a sensitive marker of cardiovascular risk, potentially closely associated with mitochondrial function decline, increased oxidative stress, and immune system alterations (34–36). Beyond these physiological mechanisms, we cannot overlook the potential statistical limitations of our research. Variations in sample sizes across different age subgroups might introduce certain uncertainties. In contrast, the AIP index demonstrated significant predictive value for cardiovascular risk in subgroups aged ≥62 years and <62 years. This finding suggests a potential systemic characteristic of lipid metabolic abnormalities that may persist across different age stages. However, these observations require further validation through large-scale prospective studies. This finding underscores the critical importance of comprehensive cardiovascular risk assessment in elderly colorectal cancer patients and provides novel perspectives for individualized risk prediction. Future research should further validate and explore the intrinsic mechanisms of this discovery by expanding sample sizes, conducting multi-center cohort studies, and integrating molecular biological investigations. Such approaches will contribute to developing more personalized cardiovascular risk management strategies for elderly colorectal cancer patients.

Our proteomic analysis identified 14 proteins that function as mediators between metabolic indices and CVD, several of which merit particular attention. DCUN1D2 participates in the neddylation protein modification pathway (37), which plays a crucial role in cardiac development and function. Li et al. demonstrated that neddylation affects protein stability and activity through various regulatory mechanisms, subsequently influencing protein transport and repair processes in cardiomyocytes (38). Previous studies have implicated PLAUR in cardiovascular disease development (39). However, our analysis revealed a negative mediating proportion (−7.7%). This suggests that PLAUR may exert inhibitory or protective regulatory effects on CVD development in specific pathological contexts (40). Interestingly, we observed that CD83 and SIGLEC10 exhibited opposite mediating effects in the AIP and TyG pathways. This discrepancy likely reflects the distinct metabolic aspects captured by each index—AIP primarily reflects lipid metabolism abnormalities (41), while TyG primarily indicates insulin resistance (42). In the context of abnormal lipid metabolism (high AIP), CD83, a key marker for dendritic cell and macrophage maturation (43), appears to exert significant immunomodulatory functions. CD83 regulates dendritic cell and macrophage polarization by inhibiting the NF-κB and ERK-STAT-1 pathways, downregulating pro-inflammatory factors such as TNF-α and IL-6, attenuating chronic low-grade inflammation triggered by lipid metabolism abnormalities, preventing small dense LDL particle formation, and protecting vascular endothelial function. Ultimately, CD83 appears to play a protective role at the metabolic-immunological interface, reducing cardiovascular risk (44). Conversely, in the high TyG group characterized by more severe metabolic disturbances and chronic inflammation, SIGLEC10 can directly bind to vascular adhesion protein-1 (VAP-1) as its substrate (45). Chen et al. showed that VAP-1 enzymatic activity produces toxic metabolites leading to vascular endothelial damage and oxidative stress, accelerating atherosclerosis and diabetes-related cardiovascular complications (46). Finally, we identified SPINK6, an understudied protein. Although SPINK6 functions as an important serine protease inhibitor in several biological processes, its relationship with cardiovascular disease requires further investigation.

Additionally, we found that the mediator proteins were primarily involved in the hematopoietic cell lineage and tight junction pathways, reflecting the influence of metabolic status on immune response and endothelial function. Previous research has shown that hematopoietic cell lineage pathways participate in pathological angiogenesis, where proinflammatory cytokines decrease vascular stability, thereby increasing cardiovascular risk (32, 47). Tight junction pathways comprise various proteins that collectively maintain endothelial cell integrity. Xu et al. demonstrated that endothelial cell damage or dysfunction leads to vascular barrier disruption, increased vascular permeability, and enhanced inflammatory cell invasion, thus accelerating atherosclerosis and other cardiovascular diseases (48). Comprehensive investigation of these mediator protein pathways offers new avenues for preventing and treating cardiovascular disease in rectal cancer patients.

From a practical standpoint, our mechanistic findings hold direct clinical significance. The TyG and AIP indices, derived from routine laboratory tests, provide a simple, noninvasive, and cost-effective tool for stratifying cardiovascular disease risk in colorectal cancer patients. Our age-stratified analysis identifies patients aged ≥62 years as a high-risk subgroup requiring enhanced cardiovascular monitoring when these indices are elevated. Integrating automated calculation of TyG and AIP into electronic health record systems with clinical decision support can facilitate the implementation of cardiovascular oncology programs. Furthermore, the identified mediating proteins represent potential therapeutic targets for precision cardiovascular prevention. Importantly, our matched analysis demonstrates that cancer-associated cardiovascular disease exhibits distinct metabolic characteristics from non-cancer cardiovascular disease, validating the necessity for cancer-specific risk models. These findings can effectively enhance the health quality of colorectal cancer survivors.

Although our study provides important novel insights, several limitations warrant careful consideration in a progressive manner: First, the retrospective nature of our study precludes definitive causal inference; however, our findings provide important clues suggesting a potential causal relationship between metabolic indices and the development of secondary cardiovascular disease in rectal cancer patients. Second, our analysis relied on single time-point measurements of TyG, AIP, and protein levels. This approach precluded the examination of dynamic changes in these biomarkers during cancer treatment and the time-dependency of their impact on CVD risk development, which may evolve throughout the disease course. Third, despite adjusting for major confounders, we could not account for individual treatment regimens due to the retrospective design. Different chemotherapy protocols, radiation doses, and surgical approaches may differentially impact both metabolic parameters and cardiovascular risk. Future prospective studies with detailed treatment data are needed to disentangle these effects. Fourth, the generalizability of our findings requires careful consideration. Since our mediator analyses were based on UKB data (predominantly European ancestry) and our clinical cohort was derived from a single tertiary care center, the applicability of our results to other ethnic populations and healthcare settings requires further validation through multi-center and multi-ethnic studies. Metabolic markers may have different prognostic thresholds across diverse populations. Fifth, our predictive models have not been externally validated in independent cohorts. External validation is a critical next step to confirm the generalizability and clinical utility of our risk stratification approach before implementation in routine clinical practice. Finally, our analysis approach faces inherent limitations regarding the complex biological interactions among the identified mediator proteins. Complex regulatory relationships likely exist between these proteins, potentially leading to overlapping effects, interplay, and covariance. These intricate molecular networks merit further investigation to fully elucidate the mechanistic pathways connecting metabolic indices to cardiovascular outcomes in rectal cancer.

## 4 Conclusions

In Conclusion, our study represents the first demonstration of significant association between TyG and AIP with secondary cardiovascular disease in rectal cancer patients, with these associations being more pronounced among patients aged over 62. Through proteomic analysis, we identified key mediator proteins connecting these metabolic indices to CVD development, notably discovering previously unexplored potential therapeutic targets including DCUN1D2, CD177, CYTH3, SPINK6, and CA11. These findings elucidate the molecular pathways underlying secondary CVD in rectal cancer, providing a foundation for developing targeted preventive strategies.

## Data Availability

The raw data supporting the conclusions of this article will be made available by the authors, without undue reservation. The UK Biobank data used in this study are available from the UK Biobank upon reasonable request (https://www.ukbiobank.ac.uk/).
