# Supplementary material for: Triglyceride–glucose index and atherogenic index of plasma as predictors of cardiovascular risk in rectal cancer survivors
Source: Front Cardiovasc Med. 2026 Mar 9;13:1707899. doi: 10.3389/fcvm.2026.1707899 (PMC13006231; doi:10.3389/fcvm.2026.1707899)
Supplement: Supplementary file 1 [file Table1.docx]

**Supplementary material**

**Supplementary Table 1.** Nine main categories of CVD in rectal cancer patients with CVD and patients with CVD.

**Supplementary Table 2.** TyG index quartile stratification and multi-model logistic regression analysis.

**Supplementary Table 3.** AIP index quartile stratification and multi-model logistic regression analysis.

**Supplementary Table 4.**Age interaction effect of TyG and AIP in CVD : a sensitivity analysis based on 60-year age threshold.

**Supplementary Table 5.** Age interaction effect of TyG and AIP in CVD : a sensitivity analysis based on 65-year age threshold.

**Supplementary Table 6.** Differences in model fitness between composite indexe and TyG and AIP.

**Supplementary Table 7.**Drug target analysis of mediator proteins based on DrugBank and PharmGKB databases.

**Supplementary Figure 1.** Mediating effects of 109 proteins in the association between secondary CVD and TyG index.

**Supplementary Figure 2.** Mediating effects of 90 proteins in the association between secondary CVD and AIP index.

**Supplementary Figure 3.** Construction of Protein-Protein Interaction Network for mediating proteins between TyG and AIP based on the STRING database.

**Supplementary Table 1.** Nine main categories of CVD in rectal cancer patients with CVD and patients with CVD.

| Categories | Rectal patients With CVD  （n=191） | Patients With CVD  （n=191） |
| --- | --- | --- |
| Coronary artery disease | 51（26.7） | 47（24.61） |
| Arrhythmia | 46（24.1） | 22（11.52） |
| Congestive heart failure | 16（8.38） | 38（19.89） |
| Valvular disease | 9（4.71） | 11（5.76） |
| Pulmonary hypertension | 4（2.1） | 16（8.38） |
| Thrombotic disease | 42（21.99） | 13（6.81） |
| Peripheral vascular disease | 12（6.29） | 27（14.14） |
| Stroke | 4（2.08） | 12（6.28） |
| Pericardial complication | 7（3.65） | 5（2.61） |

**Supplementary Table 2.** TyG index quartile stratification and multi-model logistic regression analysis.

|  | Model 1 | | | Model 2 | | | | Model 3 | |  |
| --- | --- | --- | --- | --- | --- | --- | --- | --- | --- | --- |
|  | OR(95%*CI*) | *P*-Value | OR(95%*CI*) | | *P*-Value | | OR(95%*CI*) | | *P*-Value | |
| Q1 | 1.000 |  | 1.000 | | |  | | 1.000 |  | |
| Q2 | 1.261（0.804-1.986） | 0.313 | 1.268（0.802-2.015） | | | 0.310 | | 1.289（0.814-2.049） | 0.281 | |
| Q3 | 1.581（1.024-2.460） | 0.039 | 1.595（1.012-2.530） | | | 0.045 | | 1.351（1.032-1.772） | 0.030 | |
| Q4 | 1.601（1.085-2.381） | 0.018 | 1.572（1.062-2.344） | | | 0.025 | | 1.571（1.052-2.364） | 0.028 | |

The association between TyG and secondary CVD was further assessed by dividing the TyG index into quartiles and constructing logistic regression models. In which model 1 adjusted for age and gender, model 2 adjusted for age, gender and BMI, and model 3 continued to adjust for hyperglycemia and hyperlipidemia on the basis of model 2. Results are expressed as OR and 95% confidence intervals (95% CI).

Note: 1.000 is the reference

**Supplementary Table 3.** AIP index quartile stratification and multi-model logistic regression analysis.

|  | | Model 1 | | | Model 2 | | | Model 3 | | |
| --- | --- | --- | --- | --- | --- | --- | --- | --- | --- | --- |
|  | OR(95%*CI*) | | *P*-Value | | OR(95%*CI*) | *P*-Value | | OR(95%*CI*) | *P*-Value | |
| Q1 | 1.000 | |  | 1.000 | | |  | 1.000 | |  |
| Q2 | 1.553（0.985-2.472） | | 0.060 | 1.552（0.979-2.482） | | | 0.063 | 1.555（0.981-2.488） | | 0.062 |
| Q3 | 1.721（1.100-2.723） | | 0.018 | 1.719（1.090-2.743） | | | 0.021 | 1.572（1.062-2.344） | | 0.024 |
| Q4 | 1.851（1.445-2.368） | | <0.001 | 1.817（1.451-2.274） | | | <0.001 | 1.581（1.223-2.051） | | <0.001 |

The association between AIP and secondary CVD was further assessed by dividing the AIP index into quartiles and constructing logistic regression models. In which model 1 adjusted for age and gender, model 2 adjusted for age, gender and BMI, and model 3 continued to adjust for hyperglycemia and hyperlipidemia on the basis of model 2. Results are expressed as OR and 95% confidence intervals (95% CI).

Note: 1.000 is the reference

**Supplementary Table 4.**Age interaction effect of TyG and AIP in CVD : a sensitivity analysis based on 60-year age threshold.

| Interaction term | ​​Multiplicative interaction | | Additive interaction | | |
| --- | --- | --- | --- | --- | --- |
|  | OR（95%CI） | *P*-Value | RERI（95%CI） | AP(95%CI) | SI(95%CI) |
| Age&TyG | 0.964（0.933,0.996） | 0.027 | 0.624（0.914,1.162） | 0.606（0.468,3.667） | 2.266（1.268.4.050） |
| Age&AIP | 0.985（0.971,1.000） | 0.045 | 0.926（0.864,0.993） | 0.868（0.821,0.918） | 2.082（1.308,3.314） |

**Supplementary Table 5.** Age interaction effect of TyG and AIP in CVD : a sensitivity analysis based on 65-year age threshold.

| Interaction term | ​​Multiplicative interaction | | Additive interaction | | |
| --- | --- | --- | --- | --- | --- |
|  | OR（95%CI） | *P*-Value | RERI（95%CI） | AP(95%CI) | SI(95%CI) |
| Age&TyG | 0.611（0.382,0.978） | 0.040 | 0.415（0.340,0.693） | 0.976（0.960,0.993） | 1.682（1.304,2.171） |
| Age&AIP | 0.857（0.757,0.970） | 0.015 | 0.974（0.962,0.987） | 0.703（0.259,1.905） | 0.743（0.340,1.624） |

**Supplementary Table 6.** Differences in model fitness between composite indexe and TyG and AIP.

| Model | AIC | BIC |
| --- | --- | --- |
| TyG | 1122.03 | 1132.56 |
| AIP | 1123.01 | 1133.54 |
| Composite index | 1121.66 | 1132.19 |

**Supplementary Table 7.**Drug target analysis of mediator proteins based on DrugBank and PharmGKB databases

| ​​Mediator protein​ | ​​Disease Type​ | ​​Drug Target Status​​ |
| --- | --- | --- |
| BMP6 | Dilated cardiomyopathy | Sotatercept |
| CLU | Atherosclerosis | Investigational target |
| DCUN1D2 | Atherosclerosis | - |
| CD177 | Acute myocardial infarction | - |
| CD22 | Esophageal squamous cell carcinoma | Inotuzumab ozogamicin |
| CD83 | Autoimmune diseases | Investigational target |
| CYTH3 | Metabolic disorders | - |
| SPINK6 | Influenza | - |
| LAMP3 | Endometrial cancer | Investigational target |
| PLAUR | Breast cancer | uPAR-targeting antibody |
| SIGLEC10 | Malignant glioma | Investigational target |
| CA11 | Neuroglioma | - |

Note: "- " stands for no research.


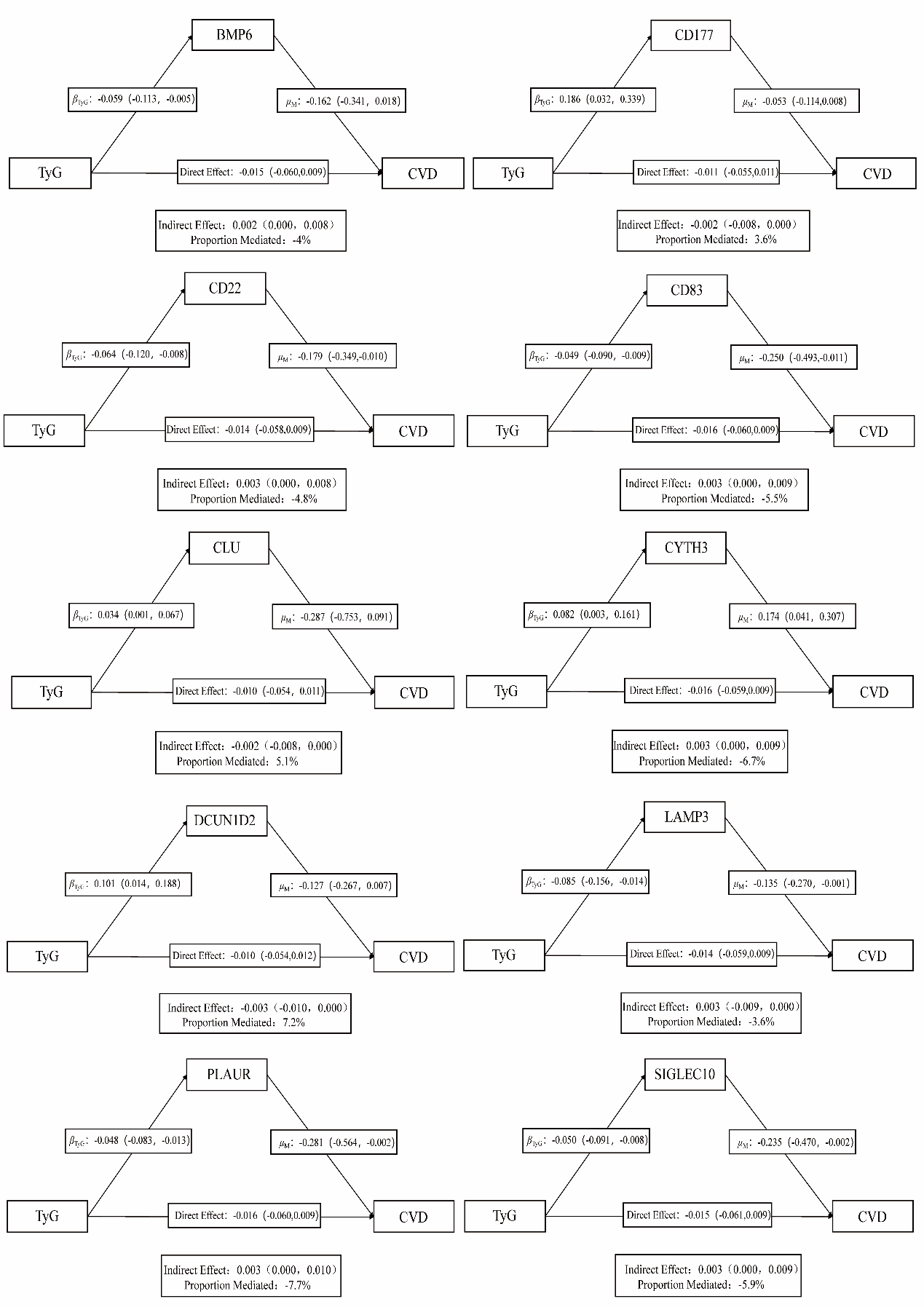


**Supplementary Figure 1.** Mediating effects of 109 proteins in the association between secondary CVD and TyG index(n=10).

For each protein, path coefficients (β) with 95% confidence intervals are shown along the arrows. Direct effect represents the association between TyG and CVD after adjusting for the mediator protein. Indirect effect indicates the product of path coefficients from TyG to protein and from protein to CVD. Proportion mediated represents the percentage of the total effect that is mediated through each protein pathway. Negative values in path coefficients indicate inverse associations.


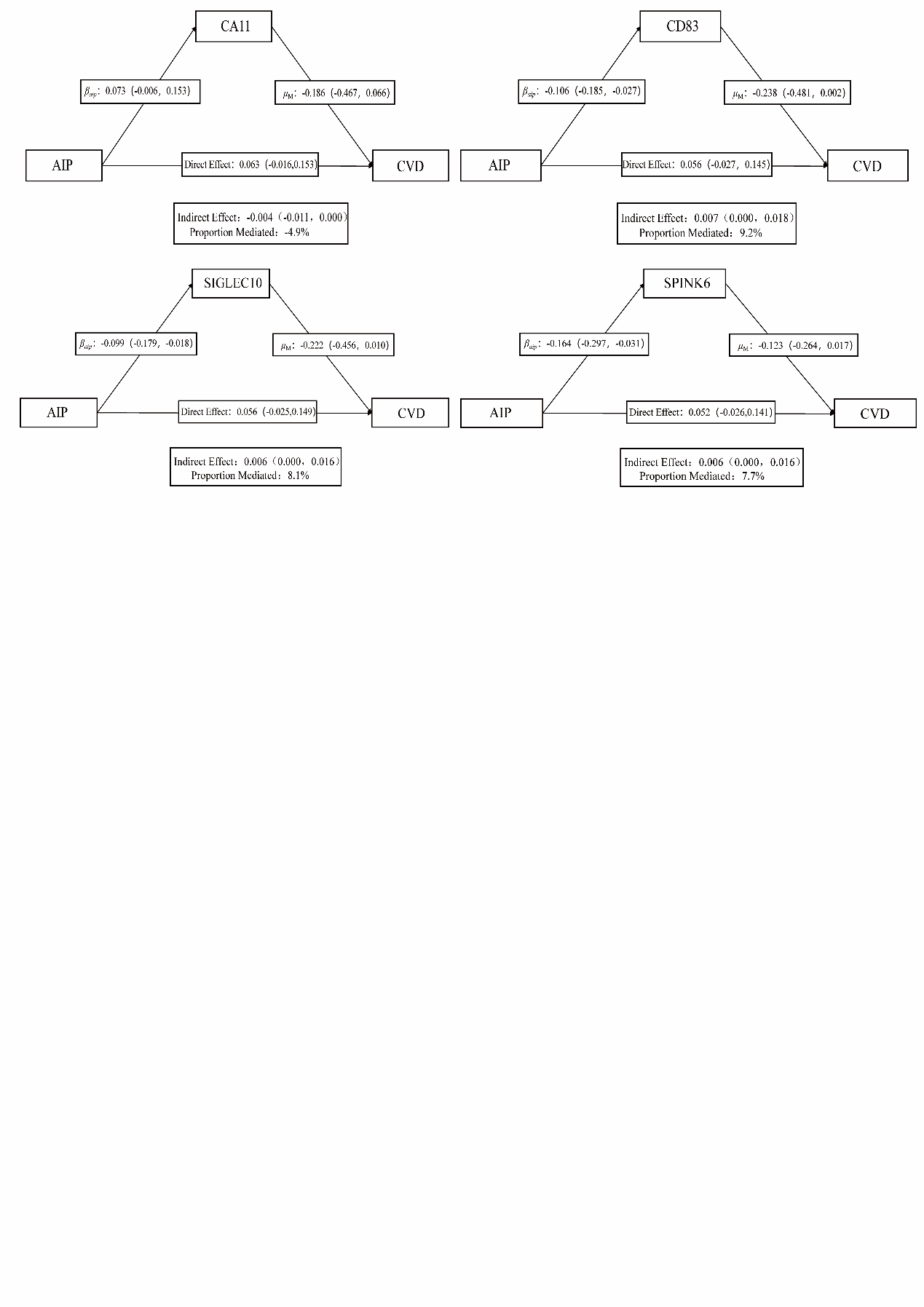


**Supplementary Figure 2.** Mediating effects of 90 proteins in the association between secondary CVD and AIP index(n=4).

For each protein, path coefficients (β) with 95% confidence intervals are shown along the arrows. Direct effect represents the association between AIP and CVD after adjusting for the mediator protein. Indirect effect indicates the product of path coefficients from AIP to protein and from protein to CVD. Proportion mediated represents the percentage of the total effect that is mediated through each protein pathway. Negative values in path coefficients indicate inverse associations.


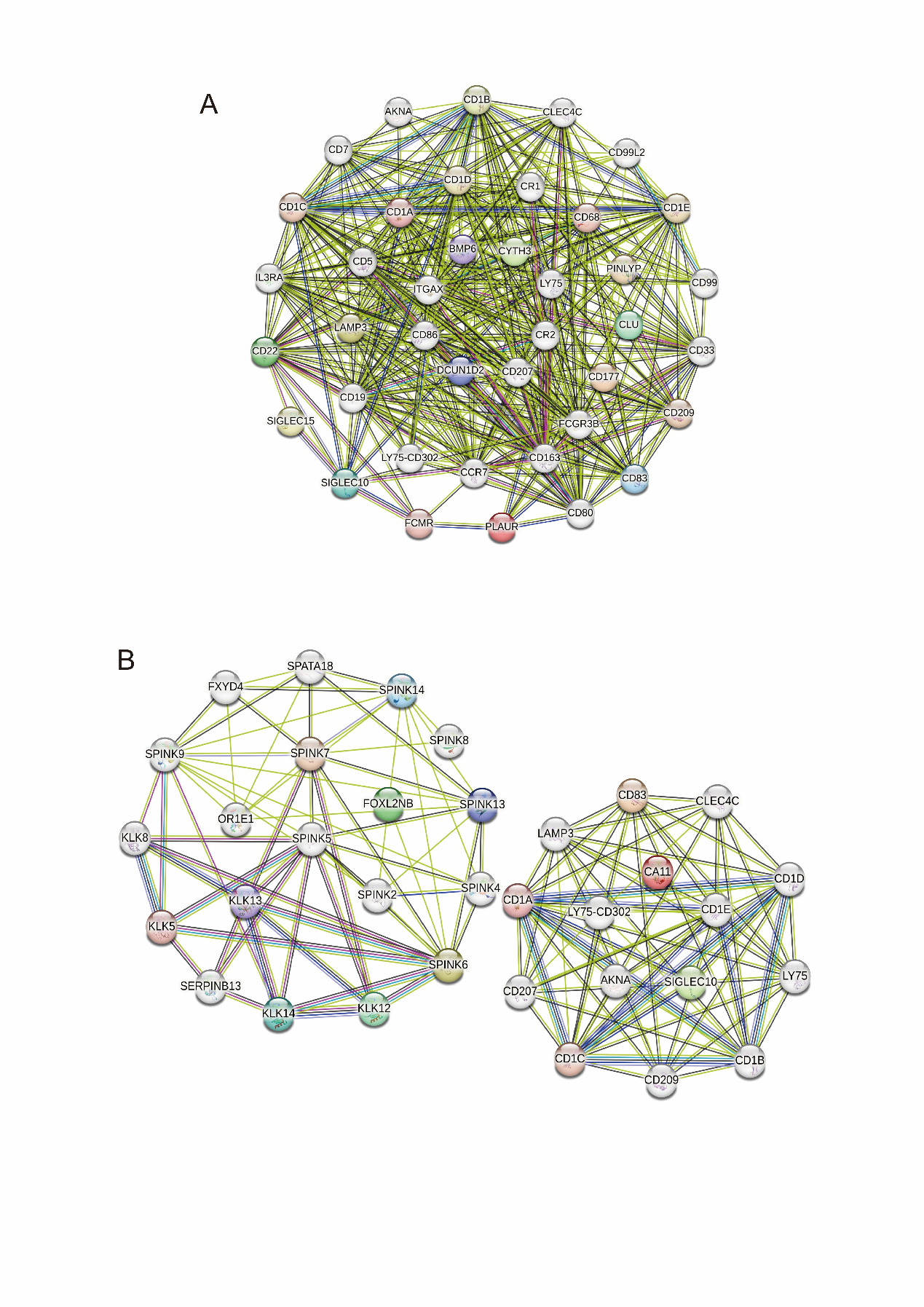


**Supplementary Figure 3.** Construction of Protein-Protein Interaction Network for mediating proteins based on the STRING database. **A.** Potential interacting proteins with TyG index-related mediator proteins(n=40). **B.** Potential interacting proteins with AIP index-related mediator proteins(n=34).

Nodes represent proteins, with colored nodes indicating query proteins and first shell of interactors, while white nodes show second shell of interactors; node fill indicates 3D structure knowledge (filled = known or predicted, empty = unknown). Edges represent specific and meaningful protein-protein associations, indicating proteins jointly contribute to a shared function. Edge colors represent evidence types: known interactions (light blue from curated databases, pink for experimentally determined), predicted interactions (green for gene neighborhood, blue for gene fusions and gene co-occurrence), and others (yellow for text mining, black for co-expression, purple for protein homology).
